# Highly Conductive Peroxidase-like Ce-MoS_2_ Nanoflowers for the Simultaneous Electrochemical Detection of Dopamine and Epinephrine

**DOI:** 10.3390/bios13121015

**Published:** 2023-12-06

**Authors:** Annadurai Thamilselvan, Thinh Viet Dang, Moon Il Kim

**Affiliations:** Department of BioNano Technology, Gachon University, 1342 Seongnamdae-ro, Sujeong-gu, Seongnam 13120, Gyeonggi, Republic of Korea; tamilselvan8033@gmail.com (A.T.); dvietthinh96@gmail.com (T.V.D.)

**Keywords:** Ce-MoS_2_ nanoflowers, electrochemical biosensors, dopamine, epinephrine, peroxidase mimic

## Abstract

The accurate and simultaneous detection of neurotransmitters, such as dopamine (DA) and epinephrine (EP), is of paramount importance in clinical diagnostic fields. Herein, we developed cerium–molybdenum disulfide nanoflowers (Ce-MoS_2_ NFs) using a simple one-pot hydrothermal method and demonstrated that they are highly conductive and exhibit significant peroxidase-mimicking activity, which was applied for the simultaneous electrochemical detection of DA and EP. Ce-MoS_2_ NFs showed a unique structure, comprising MoS_2_ NFs with divalent Ce ions. This structural design imparted a significantly enlarged surface area of 220.5 m^2^ g^−1^ with abundant active sites as well as enhanced redox properties, facilitating electron transfer and peroxidase-like catalytic action compared with bare MoS_2_ NFs without Ce incorporation. Based on these beneficial features, Ce-MoS_2_ NFs were incorporated onto a screen-printed electrode (Ce-MoS_2_ NFs/SPE), enabling the electrochemical detection of H_2_O_2_ based on their peroxidase-like activity. Ce-MoS_2_ NFs/SPE biosensors also showed distinct electrocatalytic oxidation characteristics for DA and EP, consequently yielding the highly selective, sensitive, and simultaneous detection of target DA and EP. Dynamic linear ranges for both DA and EP were determined to be 0.05~100 μM, with detection limits (S/N = 3) of 28 nM and 44 nM, respectively. This study shows the potential of hierarchically structured Ce-incorporated MoS_2_ NFs to enhance the detection performances of electrochemical biosensors, thus enabling extensive applications in healthcare, diagnostics, and environmental monitoring.

## 1. Introduction

In recent years, electrochemical detection has witnessed remarkable advancements fueled by the development of nanomaterials with extraordinary conductivity and catalytic properties [1]. These nanomaterials, often endowed with enzyme-like activities, have opened new horizons for the highly selective and sensitive detection of analytes of interest [2]. The advantageous features of nanomaterials also made it possible to simultaneously detect multiple targets, providing more comprehensive sensing information with lowered total cost and thus, expanding usability in diagnostic areas [3,4,5,6,7]. For this, many different kinds of nanomaterials composed of noble metals, metal oxides, metal sulfides, carbon with doped elements, and their hybrids, with diverse morphologies have been reported [8]. Among these, MoS_2_-based nanomaterials have emerged as a promising candidate for the construction of electrochemical biosensors since they exhibit high conductivity as well as peroxidase-like activity from their synergistic elemental composition [9,10,11]. Further research is being conducted to further engineer MoS_2_-based nanomaterials to improve their electrical and catalytic properties.

DA and EP are representative catecholamine neurotransmitters that play pivotal roles in regulating various physiological processes in the human body, including the central nervous and cardiovascular systems [12,13,14]. Aberrations in their levels are associated with a range of neurological and neuropsychiatric disorders, including Parkinson’s disease, schizophrenia, and attention deficit hyperactivity disorder (ADHD) [15,16,17]. Therefore, the reliable, selective, sensitive, and simultaneous detection of both DA and EP has garnered substantial attention for realizing their applications in the clinical field [18,19]. Conventionally, DA and EP have been analyzed using instrumentation-based methods including high-performance liquid chromatography [15,20], capillary electrophoresis [21], and mass spectrometry [22]. Enzyme-linked immunosorbent assay, which needs a microplate reader for the quantitative analysis, has also been used to produce target-specific colorimetric responses [23]. While these methods generally provide accurate and sensitive assay results, they are often laborious, time-consuming, and expensive to perform, which hinders their widespread utilization, particularly in resource-limited environments. For a more convenient detection of DA and EP, biosensors that are based on enzymes, nanozymes, and other nanomaterials having unique physicochemical or electrocatalytic properties have been reported [24,25]. They display colorimetric, fluorescent, or electrochemical signals proportional to the amount of target substances, such as DA and EP, without any sophisticated procedure or instrumentation, and thus, can be recognized as potent methods to be practically used in point-of-care testing (POCT) environments. Among them, electrochemical detection methods can exhibit higher sensitivity than those of optical ones, with simple and rapid procedures at lower cost. Thus, they have been extensively studied for detecting DA and EP [26]. Diverse nanomaterials composed of noble metals [27], metal oxides [28], and carbon [29] have been used to construct electrodes for efficient electrochemical detection of neurotransmitter molecules based on their affirmative electrocatalytic property with large surface area. Although these examples demonstrate the potential of nanomaterials, including MoS_2_-based ones, to electrochemically detect DA and EP, further engineering of nanomaterials is required to achieve higher detection performance, particularly for the simultaneous detection of neurotransmitters. 

In this regard, we developed Ce-MoS_2_ NFs as highly efficient electrocatalysts for the simultaneous detection of DA and EP. Ce-MoS_2_ NFs were synthesized simply with a one-pot hydrothermal method, yielding the successful incorporation of Ce^3+^ and Ce^4+^ ions on MoS_2_ NFs. The synthesized Ce-MoS_2_ NFs were demonstrated to exhibit both higher conductivity and peroxidase-like activity, which were much higher than those of bare MoS_2_ NFs without Ce incorporation. With the incorporation of Ce-MoS_2_ NFs on a screen-printed electrode (Ce-MoS_2_ NFs/SPE), target H_2_O_2_ and neurotransmitters including DA and EP were successfully detected. Various analytical features, including selectivity and sensitivity, were investigated. 

## 2. Experimental Section

### 2.1. Materials

Thiourea, ammonium molybdate, ethanol, cerium nitrate, dopamine hydrochloride, epinephrine, 3,3′,5,5′-tetramethylbenzidine (TMB), dimethyl sulfoxide (DMSO), phosphate-buffered saline (PBS), potassium ferricyanide, and human serum were purchased from Sigma-Aldrich (St. Louis, MO, USA). Hydrogen peroxide (H_2_O_2_, 35%) was purchased from Junsei Chemical Co. (Tokyo, Japan). Deionized water, purified using a Milli-Q Purification System (Millipore, Darmstadt, Germany), was used to prepare all solutions. PBS buffers containing 0.1 M KCl (pH 6.0) were used as electrolytes by properly mixing the solutions of Na_2_HPO_4_, NaH_2_PO_4_, and KCl. 

### 2.2. Synthesis and Characterization of MoS_2_ NFs and Ce-MoS_2_ NFs

Figure S1 illustrates the synthesis of both MoS_2_ NFs and Ce-MoS_2_ NFs using the one-pot hydrothermal method. In brief, a solution was prepared by dissolving 20 mmol of thiourea and ammonium molybdate in a 50 mL water–ethanol mixture (with a volumetric ratio of V_water_:V_ethanol_ = 5:1). The solution was stirred for 10 min, followed by the addition of 0.5 mmol of cerium nitrate with a further reaction for 10 min with stirring. The combined solution was then put into a 100 mL autoclave with a Teflon lining, and it was treated hydrothermally for 24 h at 200 °C. Following the hydrothermal process, the product was collected with centrifugation (10,000× *g*, 5 min), followed by three washes in ethanol and deionized water and overnight drying at 70 °C. For comparative purposes, MoS_2_ NFs were synthesized using the same procedure, with the exception that cerium nitrate was not included. 

An X-ray diffractometer (D/MAX-2500, Rigaku Corporation, Tokyo, Japan) was used to determine the crystal structure of the produced materials using powder X-ray diffraction (XRD). In order to investigate the nanostructured morphologies of Ce-MoS_2_ NFs, two types of microscopes were used: a scanning electron microscope (Magellan 400 Microscope, FEI Co., Eindhoven, The Netherlands) and a transmission electron microscope (FEI Tecnai, OR, USA). By combining an energy dispersive spectrometer (EDS; Elementar, Vario Macro, Langenselbold Germany) with a scanning electron microscope, elemental mapping was achieved. Using the Brunauer–Emmett–Teller (BET) method, a nitrogen physisorption study was carried out to measure the specific surface area, pore size, and pore volume (3Flex, Micromeritics, Norcross, GA, USA). The elemental composition and electronic states were examined using X-ray photoelectron spectroscopy (XPS) (Sigma Probe, Thermo Scientific, Fitchburg, WI, USA). To clarify the functional groups of the synthesized materials, Fourier transform infrared spectroscopy (FT-IR) was used with an FT-IR spectrophotometer (FT/IR-4600, JASCO, Easton, MD, USA).

### 2.3. Evaluation of the Peroxidase-like Activity of Ce-MoS_2_ NFs

The oxidation of TMB in the presence of H_2_O_2_ was measured to assess the peroxidase-like activity of Ce-MoS_2_ NFs. Ce-MoS_2_ NFs (100 µg mL^−1^) were introduced into a sodium acetate buffer (0.1 M, pH 6) containing 1 mM TMB and 2 mM H_2_O_2_ in a typical experiment. After 20 min of incubation at room temperature (RT), the supernatant was separated with centrifugation (10,000× *g*, 1 min). The absorbance at 652 nm, which is the wavelength at which TMB oxidizes, was measured using a microplate reader (Synergy H1, BioTek, Winooski, VT, USA), provided by the Center for Bionano Materials Research at Gachon University (Seongnam, Republic of Korea), to analyze the blue color evolution. Steady-state kinetic assays of Ce-MoS_2_ NFs were further conducted using TMB and H_2_O_2_ as substrates. These experiments involved systematically varying the concentrations of TMB while keeping the H_2_O_2_ concentration constant, and vice versa. The equation *v* = V_max_ × [S]/(K_m_ + [S]) was utilized to compute the kinetic parameters with the use of Lineweaver–Burk plots. In this equation, *v* represents the initial velocity, V_max_ denotes the maximal reaction velocity, [S] represents the substrate concentration, and K_m_ represents the Michaelis constant.

### 2.4. Fabrication of Ce-MoS_2_ NFs/SPE Biosensors and Electrochemical Analysis

A carbon-based SPE (DropSens, Metrohm, Oviedo, Spain) was obtained and customized with the developed Ce-MoS_2_ NFs for preparing the working electrode. In brief, a blend of 2 mg of Ce-MoS_2_ NFs was dispersed in 0.5 mL ethanol. The resulting dark suspension was subjected to 2 h of ultrasonication at RT to achieve a uniform slurry. Subsequently, this slurry was deposited onto the surface of the SPE to yield the Ce-MoS_2_ NFs/SPE biosensor. 

Electrochemical analyses were conducted using a CHI600E Potentiostat (IJ Cambria Scientific Ltd., Austin, TX, USA). A conventional three-electrode electrochemical system was used, with the Ce-MoS_2_ NFs/SPE as the working electrode. Ag/AgCl saturated in KCl served as the reference electrode, and a platinum wire was used as the counter electrode. This study used electroanalytical techniques such as cyclic voltammetry (CV) and linear sweep voltammetry (LSV) to detect H_2_O_2_ and simultaneously detect DA and EP. Electrochemical experiments were carried out by adding 50 µL of sample solutions to the Ce-MoS_2_ NFs/SPE. They included H_2_O_2_ or DA and EP at various concentrations, which were dissolved in an aqueous PBS solution (0.1 M, pH 7.0) containing 0.1 M KCl electrolyte to enhance solution conductivity and prevent ion migration. Before each test, all sample solutions were freshly prepared and stored at 4 °C until the analysis to preserve the stability of DA and EP. 

## 3. Results and Discussion

### 3.1. Synthesis of Highly-Conductive Peroxidase-like Ce-MoS_2_ NFs

**Scheme 1:**
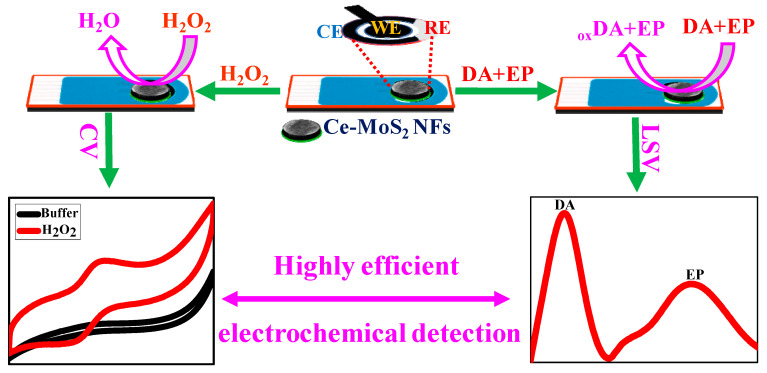
Schematic representation of Ce-MoS_2_ NF-based electrochemical detection of H_2_O_2_ or DA and EP simultaneously.

MoS_2_ NFs were proven to exhibit peroxidase-like and conductive properties [30], and herein, we hypothesized that Ce-MoS_2_ NFs, simply synthesized with one-pot hydrothermal treatment, might have higher peroxidase-like activity as well as conductivity due to the affirmative effects of Ce ions with enlarged surface area. We then incorporated Ce-MoS_2_ NFs onto the SPE to yield the Ce-MoS_2_ NFs/SPE biosensor, which successfully detected H_2_O_2_ as well as both DA and EP simultaneously, based on their high peroxidase-like activity and electrocatalytic activity, respectively. In proportion to the amount of DA and EP, characteristic oxidation peak intensity was selectively and sensitively detected, enabling the simultaneous quantification of both DA and EP (Scheme 1). 

**Figure 1:**
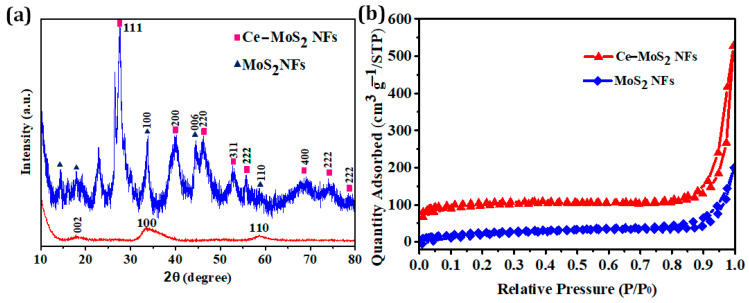
(**a**) XRD pattern and (**b**) N_2_ adsorption–desorption isotherms of Ce-MoS_2_ NFs and bare MoS_2_ NFs. In (**a**), blue and red spectra lines indicate Ce-MoS_2_ NFs and bare MoS_2_ NFs, respectively.

The structural characteristics of Ce-MoS_2_ NFs were analyzed and compared with those of bare MoS_2_ NFs without Ce incorporation. The XRD patterns of both bare MoS_2_ NFs and Ce-MoS_2_ NFs reveal the formation of hexagonal crystal phases characterized by the JCPDS card number 24-0513 (Figure 1a) [31]. The XRD patterns of bare MoS_2_ NFs precisely match the standard diffraction peaks at the (002), (100), and (110) planes. With the incorporation of Ce, the characteristic peaks related to Ce were clearly observed, such as the (110), (220), (311), (222), and (400) planes, aligning well with the JCPDS card number 81-0792 and indicating pure cubic fluorite CeO_2_ [32]. The presence of the highest valences in Ce-MoS_2_ NFs was attributed to intramolecular electron transfer occurring during synthesis [33]. Notably, the peak intensity at (002) decreases with the incorporation of Ce. This observation shows the successful incorporation of Ce into the MoS_2_ NFs, resulting in Ce-MoS_2_ NFs with a marginally modified crystal structure. The nitrogen physisorption analysis proves that the Ce-MoS_2_ NFs have a remarkably enlarged surface area of 220.5 m^2^ g^−1^, as determined using the BET method, which is notably larger than that of the bare MoS_2_ NFs (132.22 m^2^ g^−1^) (Figure 1b). The enlarged surface area of Ce-MoS_2_ NFs might play a pivotal role in enhancing their electrochemical detection performance since increasing surface area at the electrode–electrolyte interface provides a greater number of reaction sites and promotes faster diffusion kinetics. In addition, FT-IR provides clear evidence for the functional groups present in Ce-MoS_2_ NFs. The peak observed at 620 cm^−1^ is attributed to the stretching vibration of Mo-S. Multiple peaks spanning the range of 700–1150 cm^−1^ are indicative of sulfate groups, and the peaks at 3440 and 1610 cm^−1^ correspond to the stretching of O-H bonds and the bending of water (Figure S2).

**Figure 2:**
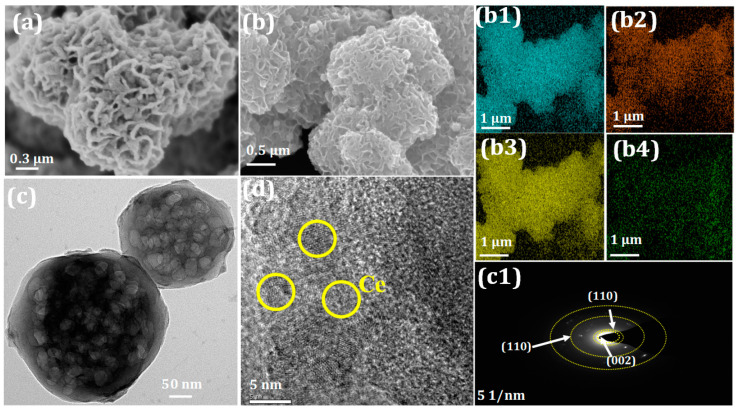
SEM of (**a**) MoS_2_ NFs and (**b**) Ce-MoS_2_ NFs, (**c**,**d**) high-resolution TEM of Ce-MoS_2_ NFs. Elemental mappings ((**b1**) Mo, (**b2**) O, (**b3**) S, and (**b4**) Ce) and selected area electron diffraction (SAED) pattern (**c1**) of Ce-MoS_2_ NFs are shown.

MoS_2_-based nanoparticles synthesized using a hydrothermal method normally tend to form sheets due to their inherent lamellar structures. These sheets subsequently aggregate, driven by van der Waals interactions, and ultimately self-assemble into three-dimensional nanostructures up to NFs. In our study, the flower-like morphology of MoS_2_ NFs was clearly observed, with diameters in ranges of 0.5 to 2 μm (Figure 2a). With the addition of cerium nitrate during the hydrothermal treatment, the flower-like morphology was still detected, and moreover, it was observed that Ce elements were uniformly distributed through the entire surface (Figure 2b). From the high-resolution TEM images, Ce ions were observed on the Ce-MoS_2_ NFs (Figure 2c,d) while showing clear diffraction rings attributed to (110), (002), and (100), all of which were also detected with the XRD analyses. 

**Figure 3:**
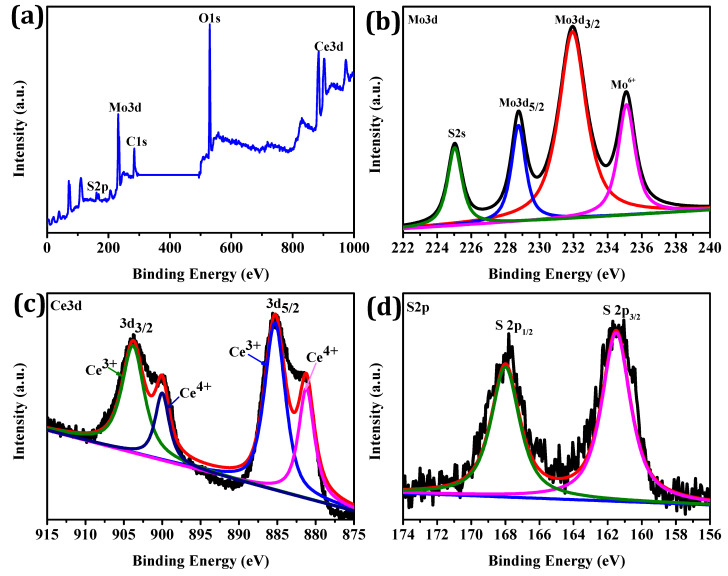
XPS spectra of (**a**) Ce-MoS_2_ NFs, (**b**) Mo 3d, (**c**) Ce 3d, and (**d**) S 2p.

The XPS analysis of Ce-MoS_2_ NFs was further performed to confirm their elemental composition and electronic states. The full survey shows that the synthesized Ce-MoS_2_ NFs are composed of Ce, Mo, S, C, and O, which is in agreement with the EDS mapping (Figure 3a). The atomic percentages of Ce and Mo in Ce-MoS_2_ NFs were determined to be ~22% and ~30%, respectively, indicating the abundance of active sites (Figure 3a). Mo 3d shows a weak satellite peak (2 s) of S species at 225.3 eV and two peaks of Mo 3d_3/2_ (232.15 eV) and Mo 3d_5/2_ (228.77 eV), which can be ascribed to Mo^4+^ (Figure 3b). The other Mo 3d_3/2_ peak observed at 235.32 eV corresponds to Mo^6+^, indicating that a certain degree of oxidation occurs due to the formation of MoO_3_. The two main characteristic peaks of Mo 3d_3/2_ and Mo 3d_5/2_ are near the meta-stable T phase of MoS_2_, which can improve the conductivity and electrocatalytic activity. The Ce 3d spectra (Figure 3c) display four distinctive peaks. The two prominent peaks correspond to Ce^3+^ 3d_5/2_, while the other two peaks are attributed to Ce^4+^ 3d_3/2_, indicating the presence of both Ce^3+^ and Ce^4+^ in the Ce-MoS_2_ NFs. The existence of Ce^4+^ can be linked to the oxidation on the surface from Ce^3+^ to Ce^4+^, due to inevitable exposure to air and moisture. Furthermore, the peaks at 162.3 eV and 163.5 eV in the S 2p spectra (Figure 3d) can be identified as the S 2p_3/2_ and S 2p_1/2_ orbitals of S^2−^, respectively. These characterizations support the successful incorporation of Ce ions onto the MoS_2_ NFs, with the multivalent electronic states helping to enhance the conductivity and electrocatalytic activity.

### 3.2. Enhanced Peroxidase-like Activity of Ce-MoS_2_ NFs

The peroxidase-like activities of Ce-MoS_2_ NFs and bare MoS_2_ NFs were evaluated based on the oxidation of TMB in the presence of H_2_O_2_, and the responses were monitored using absorption spectroscopy to analyze the corresponding blue color intensities. The results clearly show that the Ce-MoS_2_ NFs have a significant peroxidase-like activity, which is ~2-fold higher than that of the bare MoS_2_ NFs (Figure S3a,b). According to the investigations of the effects of pH on the peroxidase-like activity, pH 6 yielded the best results and was used for further studies at RT for practical convenience (Figure S3c). 

To elucidate the peroxidase-like catalytic mechanism of Ce-MoS_2_ NFs, steady-state kinetic assays were performed using TMB and H_2_O_2_ as substrates, and the resulting kinetic parameters were compared with those of HRP and previously reported peroxidase-like nanozymes including Fe_3_O_4_ nanoparticles and N-MoS_2_ NFs (Table 1, Figure S4). The apparent K_m_ values of the Ce-MoS_2_ NFs for TMB and H_2_O_2_ were 0.25 and 0.52 mM, respectively, both of which are lower than those for HRP. In particular, the K_m_ value of H_2_O_2_ was only ~14% of that of HRP and over two orders lower than that of Fe_3_O_4_ nanoparticles. This result indicates that the developed Ce-MoS_2_ NFs exhibit an excellent level of affinity toward the substrates. The enhanced substrate affinity and peroxidase-like activity of Ce-MoS_2_ NFs can be primarily attributed to the creation of additional sulfur vacancies with the incorporation of Ce elements, making higher numbers of reaction sites throughout the NFs matrices. 

### 3.3. Enhanced Electrocatalytic Performance of Ce-MoS_2_ NFs

**Figure 4:**
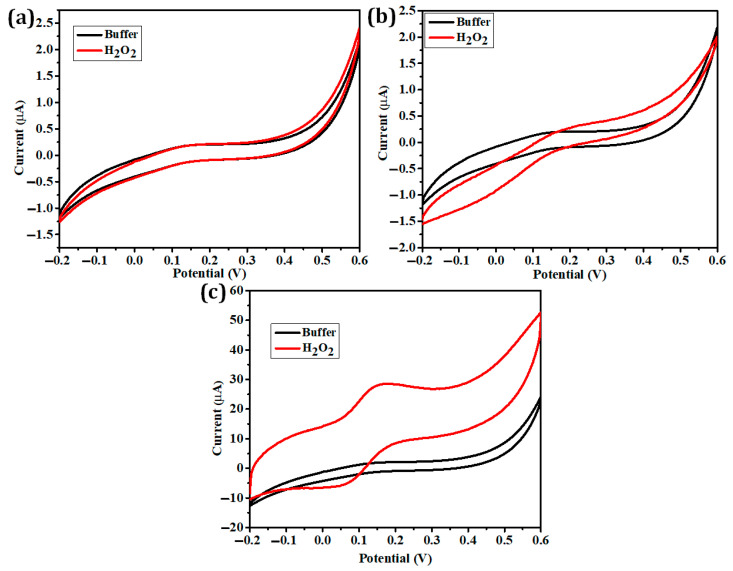
Electrochemical performances of the (**a**) bare SPE, (**b**) bare MoS_2_ NFs/SPE, and (**c**) Ce-MoS_2_ NFs/SPE in the presence (red curve) and absence (black curve) of 0.2 mM H_2_O_2_.

Based on the enhanced peroxidase-like activity of Ce-MoS_2_ NFs, the detection of H_2_O_2_ based on its electrocatalytic reduction was performed. To explore this applicability, the detection performances of the bare SPE, the bare MoS_2_ NFs/SPE, and the Ce-MoS_2_/SPE were assessed and compared in the absence and presence of H_2_O_2_ (0.2 mM) (Figure 4). The results vividly show that the Ce-MoS_2_/SPE biosensor yielded a notably enhanced reduction peak with the addition of H_2_O_2_, while the bare SPE and the bare MoS_2_ NFs/SPE showed a negligible and a significantly low peak, respectively (Figure 4), signifying the excellent electrocatalytic performance of Ce-MoS_2_ NFs toward H_2_O_2_. The difference in peak current (ΔI = I_H2O2_ − I_without H2O2_) at 0.12 V was also assessed for the above three electrodes. The ΔI for the Ce-MoS_2_/SPE reached 28.35 μA, marking over a 10-fold increase compared with that of MoS_2_/GCE. Additionally, we investigated the electrochemical performance of the Ce-MoS_2_/SPE biosensor in detecting 0.2 mM H_2_O_2_ by varying the scan rate from 10 to 100 mV s^−1^ (Figure S5). The investigation indicated that the electrocatalytic reaction followed a diffusion-controlled process, as evidenced by the relationship between peak current and scan rates. 

**Figure 5:**
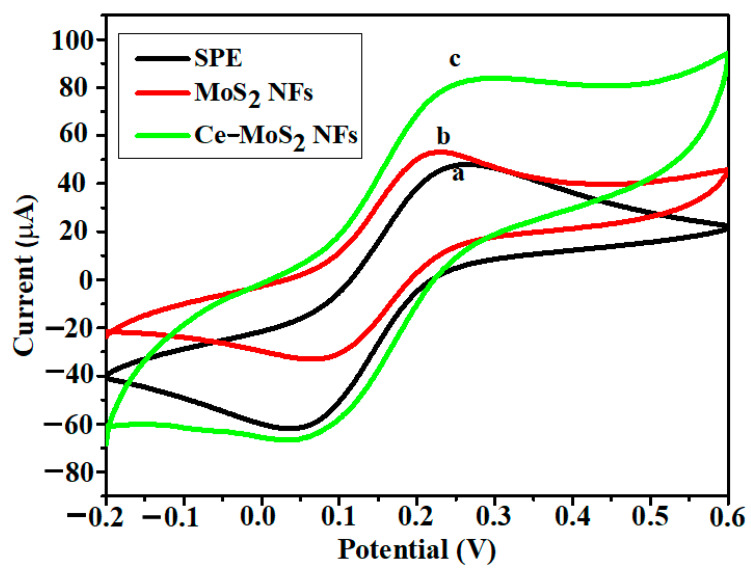
Cyclic voltammograms at a scan rate of 50 mV s^−1^ for 5 mM [K_3_Fe(CN)_6_]/[K_4_Fe(CN)_6_] depicted for the (a) bare SPE, (b) bare MoS_2_ NFs/SPE, and (c) Ce-MoS_2_ NFs/SPE.

Cyclic voltammetry (CV) was conducted on the bare SPE, the bare MoS_2_ NFs/SPE, and the Ce-MoS_2_/SPE in PBS solutions containing 0.1 M KCl and 5.0 mM [Fe(CN)_6_]^3−/4−^ as a redox probe. The sweep rate was set at 50 mV s^−1^, with scanning from −0.2 to 0.6 V. As shown in Figure 5, we observed a typical reversible redox peak for the bare SPE at 0.09 V and 0.21 V, with a peak current of 40 µA (curve a). Upon the incorporation of bare MoS_2_ NFs onto the SPE, the peak current increased to 55 µA, accompanied by an augmented ΔE_p_ of 180 mV (curve b). Conversely, when Ce-MoS_2_ NFs were introduced onto the SPE, the peak current consistently rose to 95 µA, accompanied by a reduction in ΔE_p_ by 130 mV (curve c). This significant increase in peak current in the Ce-MoS_2_ NFs/SPE configuration confirms the pivotal roles played by both MoS_2_ NFs and Ce in augmenting electrocatalytic activity and the detection current response. Moreover, Ce-MoS_2_ NFs significantly affect the material’s electrical conductivity and porosity, thereby improving its wettability, which might induce faster electron transfer at the electrode surface. The effective surface area of the Ce-MoS_2_ NF electrode can be estimated using the Randles–Sevcik equation for a reversible process observed during CV using K_3_[Fe(CN)_6_] as the oxidation–reduction probe.

I_pa_ = (2.69 × 10^5^) n^3/2^D_1/2_ *v*_1/2_ AC

Here, the effective surface area (A) is calculated using the Randles–Sevcik equation, where C represents the bulk concentration of K_3_[Fe(CN)_6_], n is the number of electrons transferred (n = 1), D is the diffusion coefficient of K_3_[Fe(CN)_6_] (measuring 7.6 × 10^−6^ cm^2^ s^−1^), and ν is the sweep rate. In this study, the effective surface area of Ce-MoS_2_ NFs was determined to be 0.83 cm^2^. Notably, this represents a substantial increase compared with the bare SPE, which has an area of 0.07 cm^2^. 

### 3.4. Simultaneous Electrochemical Detection of DA and EP Using the Ce-MoS_2_ NFs/SPE Biosensor

**Figure 6:**
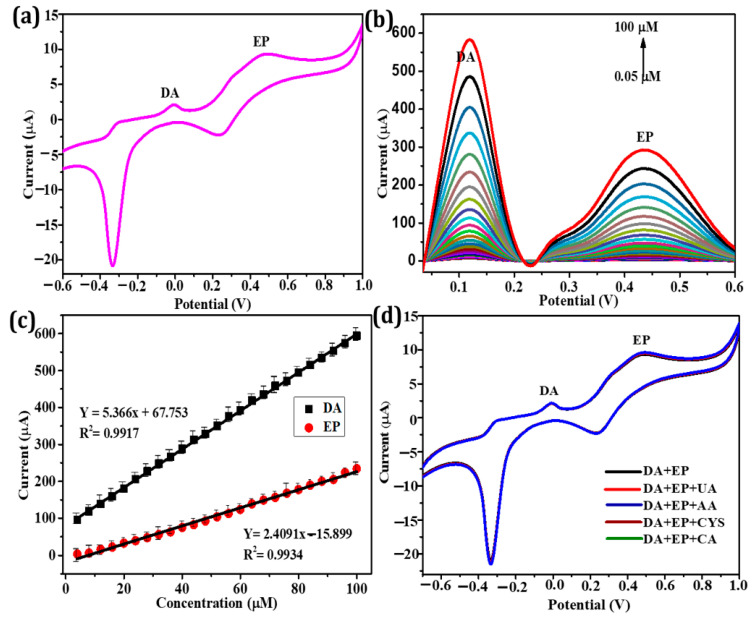
(**a**) Cyclic voltammogram, (**b**) linear sweep voltammograms, (**c**) calibration plots of current as a function of the concentrations of DA and EP, and (**d**) CV responses of DA and EP (100 nM each) in the presence of 1 μM of uric acid (UA), ascorbic acid (AA), cysteine (CYS), and critic acid (CA), using the Ce-MoS_2_ NFs/SPE biosensor.

For demonstrating the simultaneous detection capability for DA and EP using the Ce-MoS_2_ NFs/SPE biosensor, the CV of the fabricated electrodes was conducted using a sample solution containing both DA and EP. When both neurotransmitters were present together in 0.1 M KCl (pH 7.0), distinct redox peaks were observed, with a significant increase in the oxidation and reduction currents (Figure 6a). These peaks corresponded to the epinephrine–epinephrine quinone and dopamine–dopamine quinone redox couples, appearing at potentials of −0.005, 0.30, −2.28, and 0.24 V, respectively (Figure S6). These results indicate that the Ce-MoS_2_ NFs electrode exhibits the capability for the simultaneous detection of both DA and EP. In addition, individual DA or EP could be detected by producing distinct redox peaks during CV (Figure S7). 

To confirm the practical applicability of the Ce-MoS_2_ NFs/SPE biosensor for the simultaneous electrochemical detection of DA and EP using reversible processes, the LSV technique was used as the electroanalytical tool. The LSV experimental results with varying concentrations of DA and EP (ranging from 0.05 to 100 µM) are presented in Figure 6b. An evident increase in the oxidation currents corresponding to DA and EP was observed as their concentrations increased. This rise in the respective current can be attributed to the electrochemical behaviors of Ce-MoS_2_ NFs regarding the oxidation of DA and EP. The calibration plots revealed a linear relationship between the oxidation peak current and the concentrations of DA and EP within the range of 0.05 to 100 µM. The linear regression equations, I (µA) = 5.37 (DA) (µA µM^−1^) + 67.75 and I (µA) = 2.41 (EP) (µA µM^−1^) + 15.90, along with regression coefficients of over 0.99 (Figure 6c), confirm the clear linear relationship and high detection sensitivity. Calculated based on the signal-to-noise ratio (S/N = 3), the limit of detection (LOD) values for DA and EP on the Ce-MoS_2_ NFs/SPE biosensors were determined to be ~28 and 44 nM, respectively. When compared with recently reported DA and EP sensors, it becomes evident that the Ce-MoS_2_ NFs/SPE biosensors exhibited the highest level of sensitivity in the simultaneous detection of DA and EP (Table S1) [3,13,35,36,37,38,39,40,41]. The developed biosensors also showed high selectivity toward target DA and EP, while possible interfering molecules did not yield any considerable signal (Figure 6d). These investigations clearly demonstrate the significant potential of Ce-MoS_2_ NFs to construct efficient electrodes and biosensors for the simultaneous detection of both DA and EP, with sufficiently high selectivity and sensitivity. 

Reusability and storage stability are important characteristics in POCT aspects. As displayed in Figure S8, the developed biosensor efficiently retained its original detection capability for both DA and EP, during a 10-time iterative utilization and 7-day storage at RT, further supporting its practical applicability (Figure S8). To further corroborate the practical efficacy of the biosensor, it was applied to detect DA and EP in spiked human serum samples (Table S2). The measured concentrations of DA and EP were in good agreement with the spiked levels, yielding recovery rates ranging from 99.4% to 102.6%. These results indicate that the developed electrochemical biosensors could potentially be used as a reliable device for the detection of DA and EP in human serum samples. 

## 4. Conclusions

We developed highly conductive and peroxidase-like Ce-MoS_2_ NFs based on the synergistic incorporation of Ce onto MoS_2_ NFs, yielding enlarged surface area and enhanced redox electrocatalytic activity. The Ce-MoS_2_ NFs followed typical Michaelis–Menten kinetics and their K_m_ values for TMB and H_2_O_2_ were much lower than those of HRP. By incorporating the Ce-MoS_2_ NFs onto the SPE (Ce-MoS_2_ NFs/SPE), H_2_O_2_ or both DA and EP were conveniently detected by just dropping sample solutions on the biosensors. The Ce-MoS_2_ NFs/SPE enabled the successful simultaneous quantification of both DA and EP with excellent selectivity and sensitivity. Moreover, the developed biosensor exhibited excellent storage stability and reusability, as well as detection accuracy when spiked human serum samples were used. This study serves as a basis for the continued efforts toward the development of nanocomposite-incorporated electrochemical devices, which have significant potential in biosensing areas, particularly in POCT environments.

## Figures and Tables

**Table 1:** Comparison of the kinetic parameters of peroxidase-like Ce-MoS_2_ NFs for TMB and H_2_O_2_ with those of HRP and peroxidase-like nanozymes.

Catalyst	K_m_ (mM)		V_m_ (10^−8^ M s^−1^)		Ref.
	TMB	H_2_O_2_	TMB	H_2_O_2_	
HRP	0.434	3.7	10	8.71	[34]
Fe_3_O_4_ nanoparticles	0.098	154	3.44	9.78	[34]
N-MoS_2_ NFs	0.065	3.13	10.76	16.06	[2]
Ce-MoS_2_ NFs	0.25	0.52	10.5	17.81	This work

## Data Availability

Data are contained within the article.

## Supplementary Materials

The following supporting information can be downloaded at: https://www.mdpi.com/article/10.3390/bios13121015/s1, Table S1: Comparison of the electroanalytical sensitivity of the Ce-MoS_2_ NFs/SPE biosensor with those of recent DA and EP sensors; Figure S1: Schematic illustration of the synthesis of bare MoS_2_ NFs and Ce-MoS_2_ NFs; Figure S2: FT-IR spectra of the bare MoS_2_ NFs and Ce-MoS_2_ NFs; Figure S3: Evaluation of the peroxidase-like activity of (a) Ce-MoS_2_ NFs, (b) comparison of the activities of Ce-MoS_2_ NFs and the bare MoS_2_ NFs, and (c) effects of the reaction pH on catalytic activity; Figure S4: Michaelis–Menten curves of Ce-MoS_2_ NFs for (a) TMB and (b) H_2_O_2_ and (c,d) their corresponding Lineweaver–Burk plots (n = 3); Figure S5: CVs of the Ce-MoS_2_ NFs/SPE in 0.1 M PBS (pH 6.0) containing 0.2 mM H_2_O_2_ at different scan rates from 10 to 100 mV s^−1^ and their calibration plots; Figure S6: Reversible oxidation-reduction mechanism of DA and EP; Figure S7: Cyclic voltammograms of the Ce-MoS_2_ NFs/SPE at a scan rate of 100 mV s^−1^ for (a) 0.05 µM DA and (b) 0.05 µM EP; Figure S8: (a) Reusability and (b) storage stability of the Ce-MoS_2_ NFs/SPE biosensor. Excessive washing with 0.1 M PBS solution was used to evaluate reusability, and the electrode was kept in 0.1 M PBS solution at RT to evaluate storage stability; Table S2: Quantitative analysis of DA and EP spiked in human serum samples using the Ce-MoS_2_ NFs/SPE biosensor (n = 3).
