# Supplementary material for: Highly Conductive Peroxidase-like Ce-MoS2 Nanoflowers for the Simultaneous Electrochemical Detection of Dopamine and Epinephrine
Source: Biosensors (Basel). 2023 Dec 6;13(12):1015. doi: 10.3390/bios13121015 (PMC10742101; doi:10.3390/bios13121015)
Supplement: Supplementary file 1 [file biosensors-13-01015-s001.zip › biosensors-2729631-supplementary.pdf]

biosensors MDPI

Supplementary Materials

Highly Conductive Peroxidase-like Ce-MoS2 Nanoflowers for the Simultaneous Electrochemical Detection of Dopamine and Epinephrine

Annadurai Thamilselvan, Thinh Viet Dang and Moon Il Kim*

Department of BioNano Technology, Gachon University, 1342 Seongnamdae-ro, Sujeong-gu, Seongnam, Gyeonggi 13120, Republic of Korea; tamilselvan8033@gmail.com (A.T.); dvietthinh96@gmail.com (T.V.D.)

* Correspondence: moonil@gachon.ac.kr (M.I.K.); Tel.: +82-31-750-8563

Table S1. Comparison of the electroanalytical sensitivity of the Ce-MoS_2 NFs/SPE biosensor with those of recent DA and EP sensors.

Electrode material	Method	Analyte	Linear Range (μM)	LOD (nM)	Ref.
AgNP/SiO_2/GO/GCE	SWV	DA and EP	0-80	270; 260	[35]
GQD-TMSPED-AuNCs	AMP	DA and EP	0.001-4.0; 0.005-2.1	5; 1	[36]
PLA/GCE	CV	DA and EP	0.5-50; 0.8-500	300; 100	[37]
Ni-CNT/GCE	DPV	DA and EP	0.05-100.05	44; 32	[38]
GCE/Au-MSS	AMP	EP	2.0-100.0	200	[39]
CPE/Ni_3-xTe_2	SWV	DA and EP	4.0-3.1	150; 350	[3]
GCE/MWCNTCoTSP/GCE	AMP	EP	2.440-3.0	450	[40]
GCE/ Fe_3O_4@CNT-N	SWV	DA	2.50-65	50	[41]
GCE/MWCNT/CAP	AMP	DA and EP	5-115; 50-1150	180; 720	[13]
Ce-MoS_2/SPE	LSV	DA and EP	0.05-100	28; 44	This work

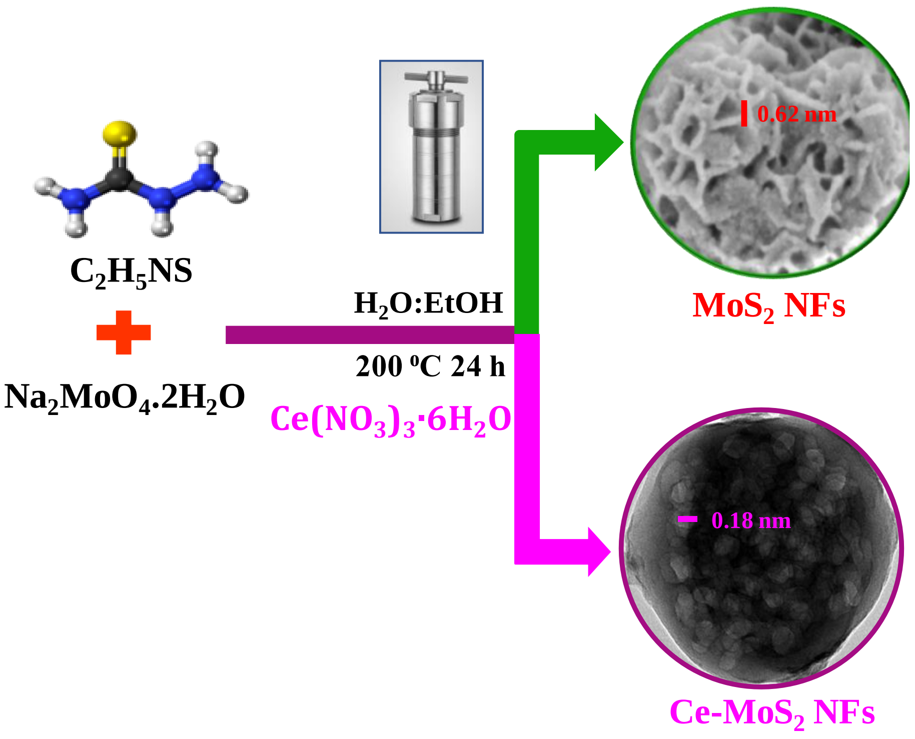

Figure S1. Schematic illustration of the synthesis of bare MoS_2 NFs and Ce-MoS_2 NFs.

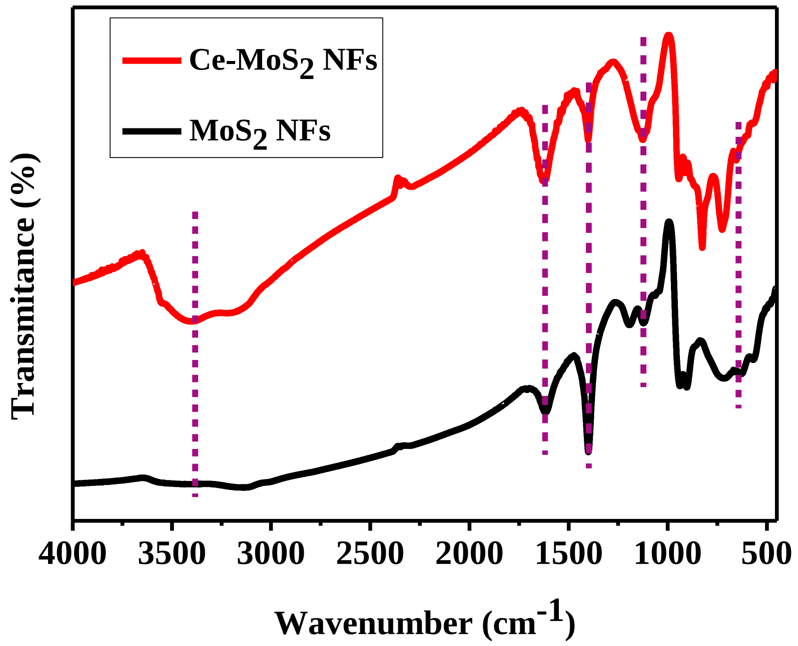

Figure S2. FT-IR spectra of the bare MoS_2 NFs and Ce-MoS_2 NFs.

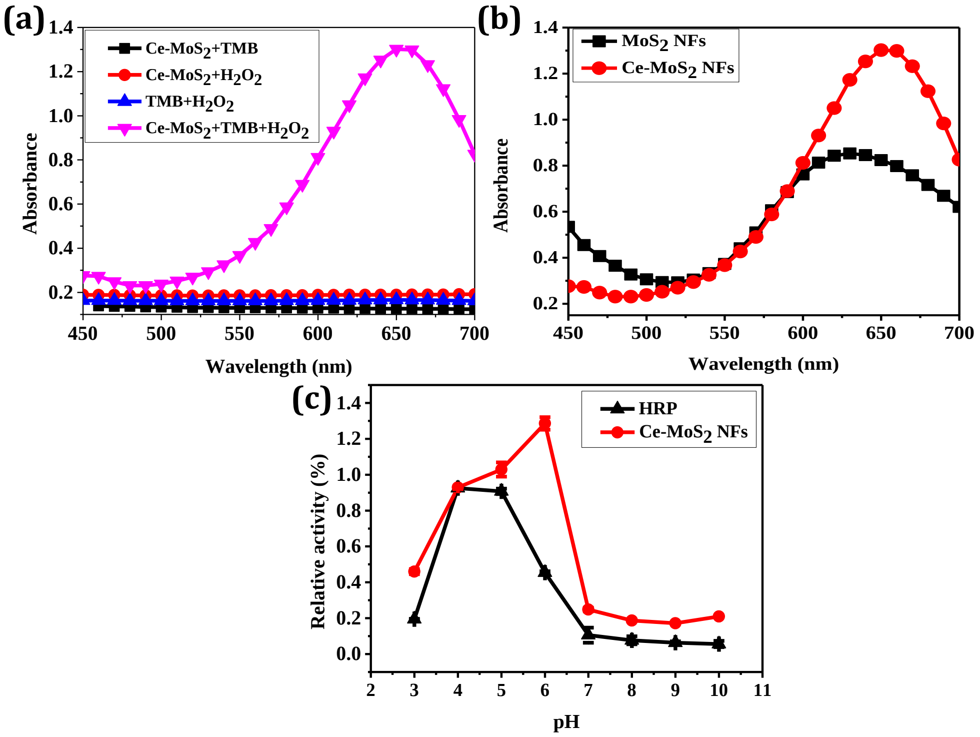

Figure S3. Evaluation of the peroxidase-like activity of (a) Ce-MoS_2 NFs, (b) comparison of the activities of Ce-MoS_2 NFs and the bare MoS_2 NFs, and (c) effects of the reaction pH on catalytic activity.

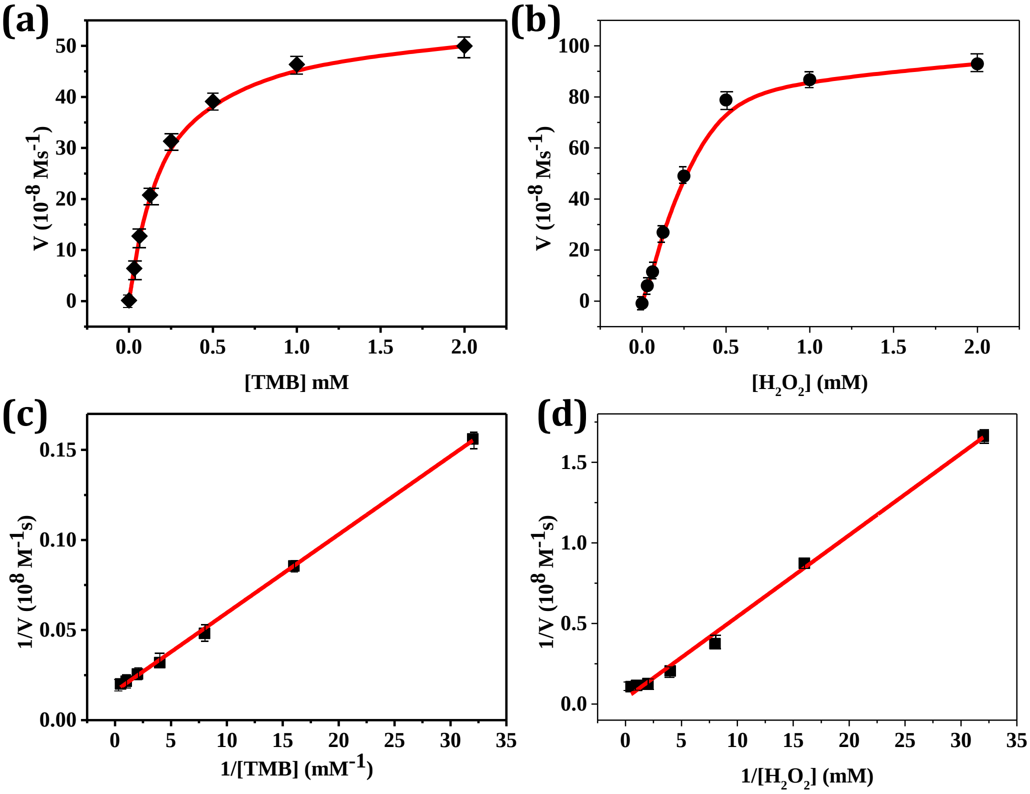

Figure S4. Michaelis–Menten curves of Ce-MoS_2 NFs for (a) TMB and (b) H_2O_2 and (c,d) their corresponding Lineweaver–Burk plots (n = 3).

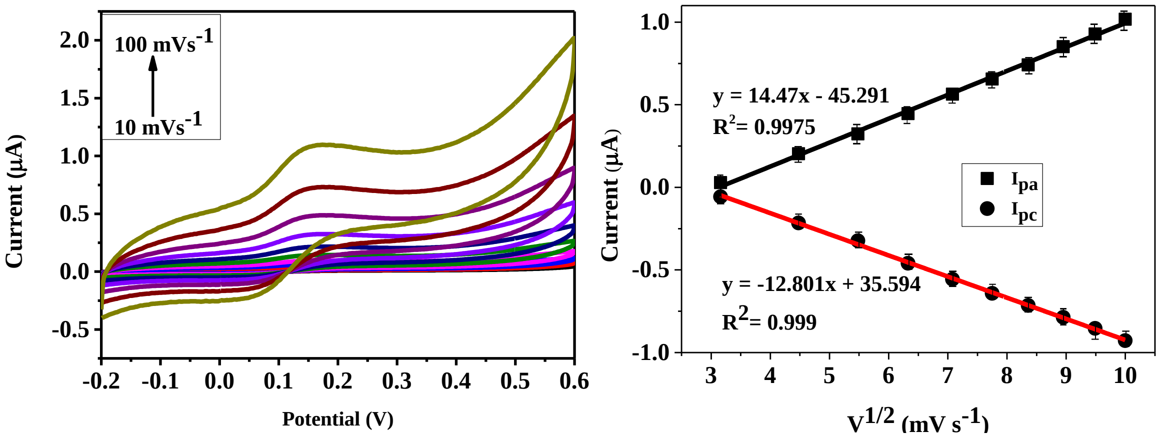

Figure S5. CVs of the Ce-MoS2 NFs/SPE in 0.1 M PBS (pH 6.0) containing 0.2 mM H_2O_2 at different scan rates from 10 to 100 mV s^{-1}, and their calibration plots.

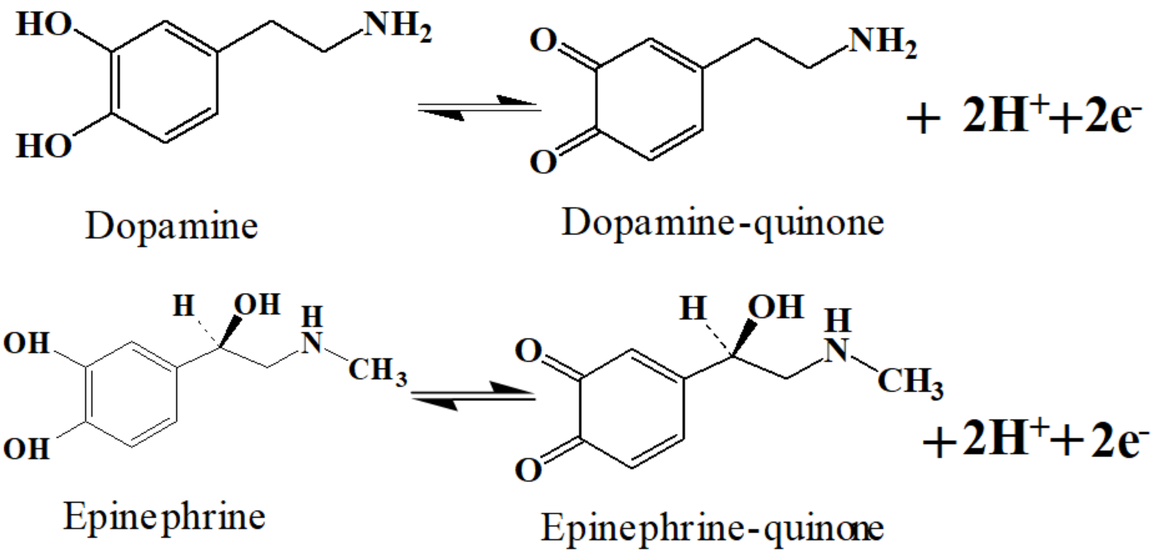

Figure S6. Reversible oxidation-reduction mechanism of DA and EP.

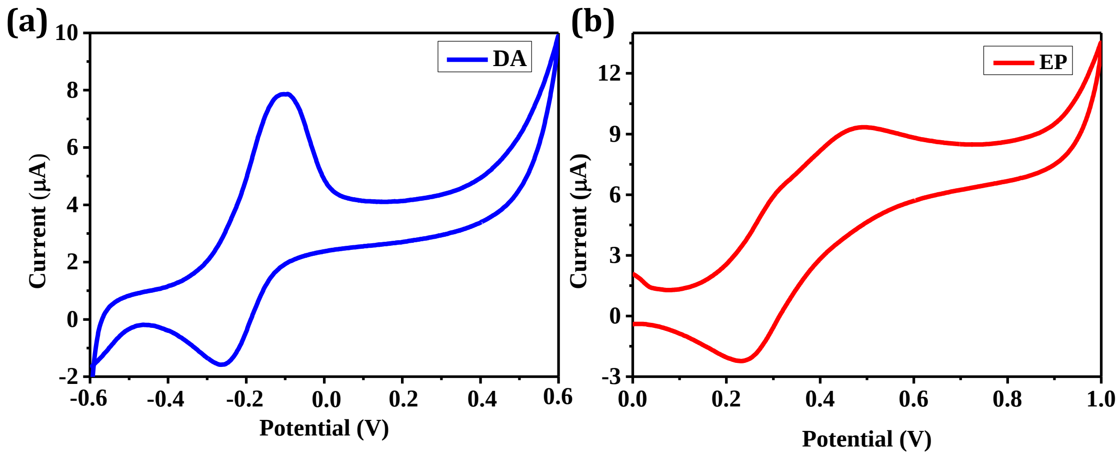

Figure S7. Cyclic voltammograms of the Ce-MoS2 NFs/SPE at a scan rate of 100 mV s^{-1} for (a) 0.05 μM DA and (b) 0.05 μM EP.

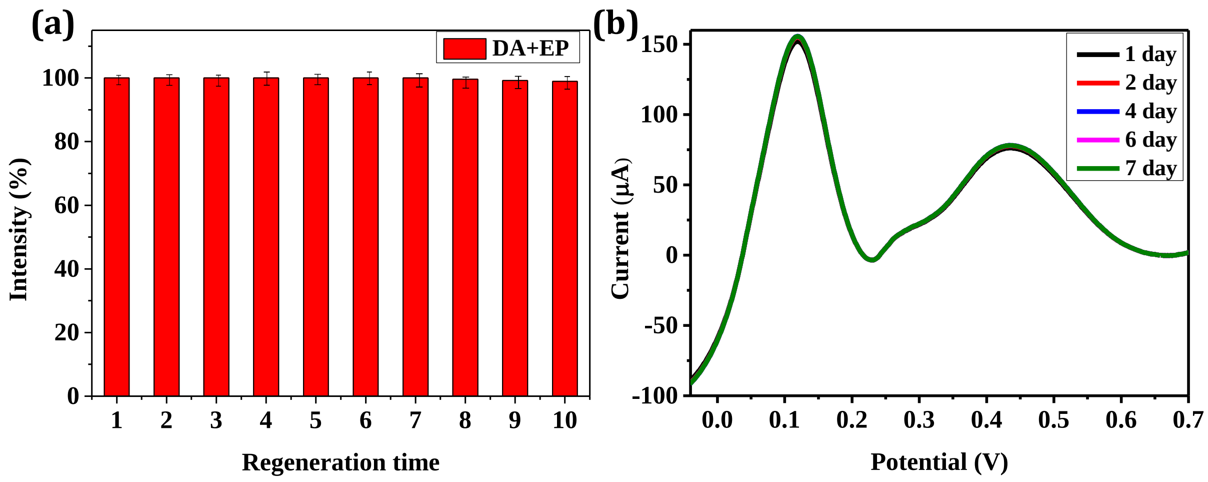

Figure S8. (a) Reusability and (b) storage stability of the Ce-MoS_2 NFs/SPE biosensor. Excessive washing with 0.1 M PBS solution was used to evaluate reusability, and the electrode was kept in 0.1 M PBS solution at RT to evaluate storage stability.

Table S2. Quantitative analysis of DA and EP spiked in human serum samples using the Ce-MoS_2 NFs/SPE biosensor (n = 3).

Sample	Added DA (μM)	Found (μM)	Recovery (%)	Added EP (μM)	Found (μM)	Recovery (%)
1	25	25.08	100.33	8	8.04	100.42
2	50	50.34	100.69	32	32.83	102.60
3	69.5	69.13	99.45	85	84.77	99.73

To assess the applicability of the developed electrochemical biosensor, the Ce-MoS_2 NFs/SPE electrodes were immersed in human serum samples, which were prepared by adding predetermined three different DA or EP were spiked. The concentrations of DA and EP in human serum samples were determined utilizing the calibration plots derived from the LSV current response. Recovery value was calculated using the equation: Recovery = (Measured value / Expected value) × 100.
